# Computation of human health risk in surface water in Ado-Odo Ota, Ogun State, Nigeria

**DOI:** 10.1016/j.dib.2018.06.051

**Published:** 2018-06-23

**Authors:** Ogbiye A. Samuel, Tenebe I. Theophilus, Emenike C. Praise God, Anake U. Winifred

**Affiliations:** a Department of Civil Engineering, Covenant University, Canaanland, Ota, Ogun State, Nigeria; b School of Water, Energy and Environment, Water Science Institute, Cranfield University, Bedfordshire, United Kingdom; c Department of Chemistry, Covenant University, Canaanland, Ota, Ogun State, Nigeria

## Abstract

Heavy metals find their way into surface and groundwater due to degrading environmental conditions, and as such consistent monitoring to avoid the adverse health implications associated with the consumption of polluted water is required. This study examined the concentrations for Lead (Pb), Nickel (Ni), Zinc (Zn), Chromium (Cr), Cadmium (Cd), Copper (Cu) and Arsenic (As) in the Surface water of River Balogun in Ota, Ogun State, Nigeria during the wet season and estimated the human health risk resulting from prolonged consumption by children and adult of dissimilar age groups without treatment. Although there were persistent occurrence of Nickel (Ni), Copper (Cu), Zinc (Zn) and Arsenic (As) in all stations sampled, the health risk assessment conducted revealed that both population groups are more likely to be affected by high concentration levels of Arsenic than any other Heavy metal present.

**Specifications Table**

**Table:** 

Subject area	Water Resources and Environmental Engineering
More specific subject area	Surface Water Quality and health-risk assessment
Type of data	Tables and figure
How data was acquired	River visits, Samples were collected during the wet season into a high density polyethene containers, ionic concentration analysis using standard methods [1], Inductively coupled plasma optical emission spectrophotometer (ICP-OES) for metal detection.
Data format	Filtered, analyzed
Experimental factors	Measuring the values of heavy metal ion content of surface water samples. Calculating the human health risk assessment after the concentration of heavy metals were obtained.
Experimental features	Determining the possible concentration levels of some selected Heavy Metals in River water samples at specific points were inhabitants collect water for various uses. Samples collected were preserved as stipulated by standard. After which, proper analysis was carried out.
Data source location	Adodo-Ota, Ogun State, Nigeria. Latitude 6°40′58.52′′N-6°41′23.92′′N and Longitude 3°8′53.87′′E - 3°8′57.86′′E.
Related research article	The data are available with this article

**Value of the data**

● The presence of Heavy Metals in surface water is unavoidable especially with the growing concerns of indiscriminate release of untreated effluent by industries within the study area. To this end, there are likelihood of adverse effects on Humans when consumed either in little or large quantities. This data obtained revealed the contamination levels of some selected Heavy Metals.
● The associated health risk is pertinent considering various means by which these Heavy Metals find their way into the body.
● The associated health risk for different age groups and population is required to estimate the at-risk groups among them for proper intervention from both Governmental and non-Governmental Organizations.
● The data is required for the design and implementation of essential and accurate treatment technique(s) for industrial effluents as well as agrochemicals that might have polluted the river.

## 1 Data

**Fig. 1:**
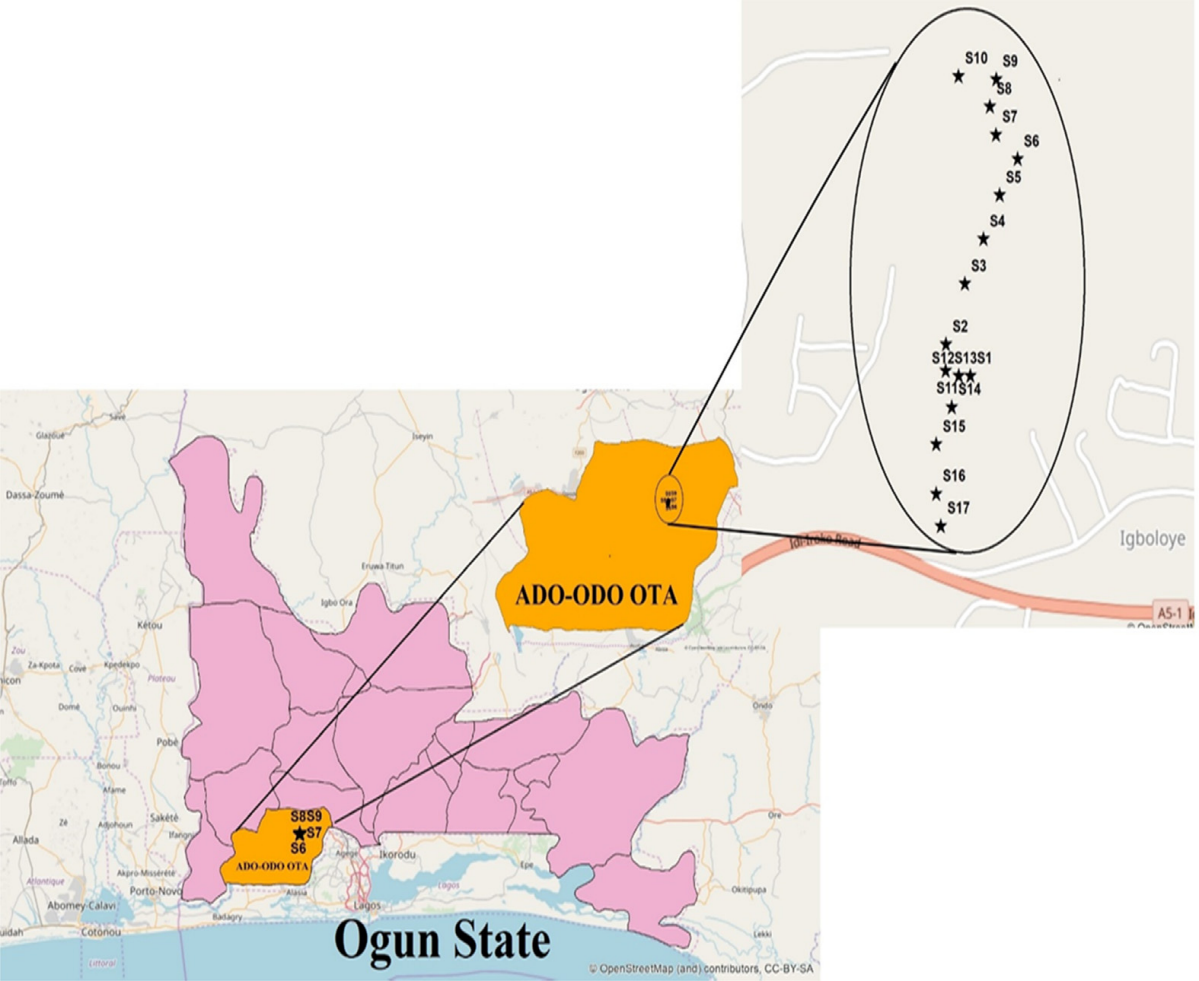
Map of study area showing sampling locations.

The data presented showed the concentration levels of selected surface water quality obtained from River Balogun in Adodo-Ota, Ogun State and the associated health risk due to oral consumption only. The presence of these heavy metals emerged due to presence of many industries situated close to the river and consistently discharged untreated liquid waste into the river under study which affected the water quality adversely [2]. This calls for investigations of both the dispersive properties and toxicity levels of these contaminants as the river were mostly utilized by dwellers downstream unconsciously [3]. Fig. 1 exposes several points along the river where samples were collected with locations between latitude 6°40′58.52′′N–6°41′23.92′′N and longitude 3°8′53.87′′E–3°8′57.86′′E, having an area of 1460 km^2^ [4].

## 2 Experimental design, materials, and methods

Seventeen (17) sampling points were assessed and a total of fifty-one samples were collected. The samples were preserved with acid and kept in a cooler to prevent speciation of the inherent metals. Thereafter, the mean values obtained from each sampling stations were used to calculate the associated health risk and comparison with standard values to ascertain whether or not these values were in concentrations below or above limits set by World Health Organization [5].

In this study, samples collected were obtained from sections of the river where inhabitants of the locality get water for various uses especially farmers. Additionally, some essential physicochemical properties of water such as pH, Total dissolved solids (TDS), Electrical Conductivity (EC) and Temperature were measured using HANNA – HI2030 device before taking collected samples for heavy metal analysis. Other heavy metals reported in this study were achieved using the Inductively coupled plasma optical emission spectrophotometer (ICP-OES). The raw values obtained from the analysis of the sampled surface water are presented in Table 1.

**Table 1:** Individual metal concentration of analyzed river water samples (*N*^d^=51 from 17 different locations).

*Station ID*	Cu (mg/L)	Zn (mg/L)	Ni (mg/L)	Cr (mg/L)	Cd (mg/L)	Pb (mg/L)	As (mg/L)
ST1	0.08	0.20	0.03	ND	ND	0.00	0.04
ST2	0.08	0.11	0.03	ND	ND	0.00	0.04
ST3	0.07	0.10	0.03	ND	ND	0.00	0.05
ST4	0.06	0.23	0.01	ND	ND	0.01	0.05
ST5	0.05	0.21	0.01	ND	ND	0.01	0.05
ST6	0.05	0.24	0.01	ND	ND	0.01	0.05
ST7	0.06	0.14	0.01	ND	ND	0.00	0.04
ST8	0.06	0.22	0.01	ND	ND	0.00	0.03
ST9	0.06	0.13	0.01	ND	ND	0.00	0.03
ST10	0.06	0.33	0.02	ND	ND	0.00	0.03
ST11	0.06	0.79	0.02	ND	ND	0.00	0.03
ST12	0.06	0.29	0.02	ND	ND	0.00	0.05
ST13	0.07	0.44	0.02	ND	ND	0.00	0.03
ST14	0.07	0.51	0.02	ND	ND	0.00	0.04
ST15	0.07	0.39	0.03	ND	ND	0.00	0.06
ST16	0.07	0.53	0.03	ND	ND	0.00	0.06
ST17	0.07	0.30	0.03	ND	ND	0.00	0.08
ST18	0.05	0.45	0.01	ND	ND	0.01	0.05
ST19	0.05	0.53	0.01	ND	ND	0.01	0.05

Consequently, these values obtained (Table 1) can be used to determine the consumption of these metals on a daily basis. In addition, the overall daily consumption or accumulation were determined with other variables known to be key parameters for the calculation of risk due to ingestion of contaminated water from this region. For instance, the concentration of various elements used for this analysis were obtained from estimating the level of contamination in water by laboratory analysis (Cfw), while the ingestion rate (IRw), frequency at which the individual is expected to be exposed to these contaminants (EFr), duration of exposure (ED), body weight (BW) and average time (ATr) were varied for children to adulthood from 6 to 12 months, 6 to 11 years, 11 to 16 years, 16 to 18 years, 18 to 21 years, ≥ 21 years and above 65 years [6]. Specifically, ingestion rate (IRw) values in L/day were 1, 1.32, 1.82, 1.78, 2.34, 2.94 and 2.73 for the different age groups mentioned respectively [6], Exposure frequency (EFr) measured in days/year were constant at 365 for all age groups [6], Exposure duration (ED) in years were constant at 6 for the first four age groups and also constant at 20 for the last three age groups [7]. Another parameter that varied as explained were the body weight of the different categories. The body weight (kg) varied at 9.1, 29.3, 54.2, 67.6, 67.6, 78.8 and 80 [6] and finally, average time (ATr) in Days was constant at 2190 for the first four age groups and also constant at 7300 for the last three age groups as well [6].The values presented in Table 2 are in accordance with laid down models or equations approved by United States Environmental Protection Agency (USEPA) and have been used in several studies in the literature [6], [8], [9], [10], [11] for estimating the chronic daily dose (CDD) or average daily dose (ADD) of heavy metals measured in mg/kg/day. The values obtained through laboratory analysis (see Table 1) are inputted into Eqs. (1) and (2) to estimate the associated risk from the consumption of surface water from River Balogun through ingestion route with focus on children and adult [12], [13] of diverse age groups resulting from variability in body mass index. This was required because different body weight are susceptible to different risk intensity [9]

(1) $$ADD_{IN}=\frac{C_{fw}\times IR_{w}\times EF_{r}\times ED}{BW\times AT_{r}}$$

**Table 2:** Oral reference dose (RfD).

Metals	RfD ingestion (mg/kg/day)
Arsenic (As)	3.00E−4
Copper (Cu)	3.70E−2
Nickel (Ni)	2.00E−2
Zinc (Zn)	3.00E−01
Lead (Pb)	3.50E−03

^*^NA=Not Applicable.

Specifically, Table 2, Table 3a, Table 3b, Table 4a, Table 4b, Table 5a, Table 5b, Table 6a(a) revealed the CDD ingestion values for the metals analyzed which were estimated with Eq. (1) while Table 2, Table 3a, Table 3b, Table 4a, Table 4b, Table 5a, Table 5b, Table 6a(b) showed the potentiality of a risk for the different population at different age groups overtime which were also estimated using Eq. (2) [14].

(2) $$HRI_{IN}=\frac{ADD_{IN}}{RfD_{metal}}$$

**Table 3a:** Average daily dose (ADD) via ingestion pathway for Cu concentration.

**Cu ADD**_**IN**_**values**							
*Station ID*	(6–12 months)	(6–11 years)	(11–16 years)	(16–18 years)	(18–21 years)	(≥21 years)	(>65 years)
ST1	8.791	3.604	2.686	2.107	2.769	2.985	2.730
ST2	8.462	3.469	2.586	2.028	2.665	2.873	2.628
ST3	8.132	3.334	2.485	1.949	2.562	2.761	2.525
ST4	6.264	2.568	1.914	1.501	1.973	2.127	1.945
ST5	5.934	2.433	1.813	1.422	1.869	2.015	1.843
ST6	5.934	2.433	1.813	1.422	1.869	2.015	1.843
ST7	6.154	2.523	1.880	1.475	1.938	2.089	1.911
ST8	6.264	2.568	1.914	1.501	1.973	2.127	1.945
ST9	6.374	2.613	1.948	1.527	2.008	2.164	1.979
ST10	6.484	2.658	1.981	1.554	2.042	2.201	2.013
ST11	6.593	2.703	2.015	1.580	2.077	2.239	2.048
ST12	6.923	2.838	2.115	1.659	2.181	2.351	2.150
ST13	7.143	2.928	2.183	1.712	2.250	2.425	2.218
ST14	7.143	2.928	2.183	1.712	2.250	2.425	2.218
ST15	7.253	2.973	2.216	1.738	2.285	2.462	2.252
ST16	7.473	3.063	2.283	1.791	2.354	2.537	2.321
ST17	7.363	3.018	2.250	1.764	2.319	2.500	2.286

**Table 3b:** Health Risk Index (HRI) via ingestion pathway for Cu concentration.

**Cu HRI**_**IN**_**values**							
*Station ID*	(6–12 months)	(6–11 years)	(11–16 years)	(16–18 years)	(18–21 years)	(≥21 years)	(>65 years)
ST1	9.010E−02	6.716E−02	5.266E−02	6.923E−02	7.462E−02	2.198E−01	6.825E−02
ST2	8.672E−02	6.464E−02	5.069E−02	6.663E−02	7.182E−02	2.115E−01	6.569E−02
ST3	8.334E−02	6.212E−02	4.871E−02	6.404E−02	6.902E−02	2.033E−01	6.313E−02
ST4	6.420E−02	4.785E−02	3.752E−02	4.933E−02	5.317E−02	1.566E−01	4.863E−02
ST5	6.082E−02	4.533E−02	3.555E−02	4.673E−02	5.037E−02	1.484E−01	4.607E−02
ST6	6.082E−02	4.533E−02	3.555E−02	4.673E−02	5.037E−02	1.484E−01	4.607E−02
ST7	6.307E−02	4.701E−02	3.686E−02	4.846E−02	5.223E−02	1.538E−01	4.778E−02
ST8	6.420E−02	4.785E−02	3.752E−02	4.933E−02	5.317E−02	1.566E−01	4.863E−02
ST9	6.532E−02	4.869E−02	3.818E−02	5.019E−02	5.410E−02	1.593E−01	4.948E−02
ST10	6.645E−02	4.953E−02	3.884E−02	5.106E−02	5.503E−02	1.621E−01	5.033E−02
ST11	6.758E−02	5.037E−02	3.950E−02	5.192E−02	5.596E−02	1.648E−01	5.119E−02
ST12	7.096E−02	5.289E−02	4.147E−02	5.452E−02	5.876E−02	1.731E−01	5.375E−02
ST13	7.321E−02	5.457E−02	4.279E−02	5.625E−02	6.063E−02	1.786E−01	5.545E−02
ST14	7.321E−02	5.457E−02	4.279E−02	5.625E−02	6.063E−02	1.786E−01	5.545E−02
ST15	7.433E−02	5.541E−02	4.345E−02	5.712E−02	6.156E−02	1.813E−01	5.631E−02
ST16	7.659E−02	5.708E−02	4.476E−02	5.885E−02	6.343E−02	1.868E−01	5.801E−02
ST17	7.546E−02	5.625E−02	4.411E−02	5.798E−02	6.249E−02	1.841E−01	5.716E−02

**Table 4a:** Average daily dose (ADD) via ingestion pathway for Zn concentration.

**Zn ADD**_**IN**_**values**							
*Station ID*	(6–12 months)	(6–11 years)	(11–16 years)	(16–18 years)	(18–21 years)	(≥21 years)	(>65 years)
ST1	21.841	8.954	6.674	5.233	6.880	7.415	6.782
ST2	11.991	4.916	3.664	2.873	3.777	4.071	3.724
ST3	10.773	4.416	3.292	2.581	3.393	3.657	3.345
ST4	25.581	10.487	7.817	6.130	8.058	8.685	7.944
ST5	23.085	9.464	7.054	5.531	7.272	7.838	7.169
ST6	26.833	11.001	8.199	6.430	8.452	9.110	8.333
ST7	15.664	6.422	4.786	3.753	4.934	5.318	4.864
ST8	24.331	9.975	7.435	5.830	7.664	8.261	7.556
ST9	14.437	5.919	4.412	3.459	4.548	4.902	4.483
ST10	36.703	15.047	11.215	8.795	11.561	12.461	11.398
ST11	86.428	35.433	26.410	20.710	27.225	29.344	26.839
ST12	31.693	12.993	9.684	7.594	9.983	10.760	9.842
ST13	48.132	19.732	14.708	11.533	15.162	16.342	14.947
ST14	55.871	22.905	17.073	13.388	17.599	18.969	17.350
ST15	43.026	17.639	13.147	10.310	13.553	14.608	13.361
ST16	58.472	23.972	17.867	14.011	18.419	19.852	18.158
ST17	32.941	13.505	10.066	7.893	10.377	11.184	10.230

**Table 4b:** Health Risk Index (HRI) via ingestion pathway for Zn concentration.

**Zn HRI**_**IN**_**values**							
*Station ID*	(6–12 months)	(6–11 years)	(11–16 years)	(16–18 years)	(18–21 years)	(≥21 years)	(>65 years)
ST1	2.985E−02	2.225E−02	1.744E−02	2.293E−02	2.472E−02	7.280E−02	2.261E−02
ST2	1.639E−02	1.221E−02	9.578E−03	1.259E−02	1.357E−02	3.997E−02	1.241E−02
ST3	1.472E−02	1.097E−02	8.604E−03	1.131E−02	1.219E−02	3.591E−02	1.115E−02
ST4	3.496E−02	2.606E−02	2.043E−02	2.686E−02	2.895E−02	8.527E−02	2.648E−02
ST5	3.155E−02	2.351E−02	1.844E−02	2.424E−02	2.613E−02	7.695E−02	2.390E−02
ST6	3.667E−02	2.733E−02	2.143E−02	2.817E−02	3.037E−02	8.944E−02	2.778E−02
ST7	2.141E−02	1.595E−02	1.251E−02	1.645E−02	1.773E−02	5.221E−02	1.621E−02
ST8	3.325E−02	2.478E−02	1.943E−02	2.555E−02	2.754E−02	8.110E−02	2.519E−02
ST9	1.973E−02	1.471E−02	1.153E−02	1.516E−02	1.634E−02	4.812E−02	1.494E−02
ST10	5.016E−02	3.738E−02	2.932E−02	3.854E−02	4.154E−02	1.223E−01	3.799E−02
ST11	1.181E−01	8.803E−02	6.903E−02	9.075E−02	9.781E−02	2.881E−01	8.946E−02
ST12	4.331E−02	3.228E−02	2.531E−02	3.328E−02	3.587E−02	1.056E−01	3.281E−02
ST13	6.577E−02	4.903E−02	3.844E−02	5.054E−02	5.447E−02	1.604E−01	4.982E−02
ST14	7.635E−02	5.691E−02	4.463E−02	5.866E−02	6.323E−02	1.862E−01	5.783E−02
ST15	5.880E−02	4.382E−02	3.437E−02	4.518E−02	4.869E−02	1.434E−01	4.454E−02
ST16	7.991E−02	5.956E−02	4.670E−02	6.140E−02	6.617E−02	1.949E−01	6.053E−02
ST17	4.502E−02	3.355E−02	2.631E−02	3.459E−02	3.728E−02	1.098E−01	3.410E−02

**Table 5a:** Average daily dose (ADD) via ingestion pathway for Ni concentration.

**Ni ADD**_**IN**_**values**							
*Station ID*	(6–12 months)	(6–11 years)	(11–16 years)	(16–18 years)	(18–21 years)	(≥21 years)	(>65 years)
ST1	2.747	1.126	0.839	0.658	0.865	0.933	0.853
ST2	3.187	1.306	0.974	0.764	1.004	1.082	0.990
ST3	3.516	1.442	1.075	0.843	1.108	1.194	1.092
ST4	0.549	0.225	0.168	0.132	0.173	0.187	0.171
ST5	0.659	0.270	0.201	0.158	0.208	0.224	0.205
ST6	0.769	0.315	0.235	0.184	0.242	0.261	0.239
ST7	1.099	0.451	0.336	0.263	0.346	0.373	0.341
ST8	1.319	0.541	0.403	0.316	0.415	0.448	0.410
ST9	1.538	0.631	0.470	0.369	0.485	0.522	0.478
ST10	1.758	0.721	0.537	0.421	0.554	0.597	0.546
ST11	1.978	0.811	0.604	0.474	0.623	0.672	0.614
ST12	2.198	0.901	0.672	0.527	0.692	0.746	0.683
ST13	2.527	1.036	0.772	0.606	0.796	0.858	0.785
ST14	2.637	1.081	0.806	0.632	0.831	0.895	0.819
ST15	3.077	1.261	0.940	0.737	0.969	1.045	0.956
ST16	3.297	1.352	1.007	0.790	1.038	1.119	1.024
ST17	3.516	1.442	1.075	0.843	1.108	1.194	1.092

**Table 5b:** Health Risk Index (HRI) via ingestion pathway for Ni concentration.

**Ni HRI**_**IN**_**values**							
*Station ID*	(6–12 months)	(6–11 years)	(11–16 years)	(16–18 years)	(18–21 years)	(≥21 years)	(>65 years)
ST1	5.631E−02	4.197E−02	3.291E−02	4.327E−02	4.664E−02	1.374E−01	4.266E−02
ST2	6.532E−02	4.869E−02	3.818E−02	5.019E−02	5.410E−02	1.593E−01	4.948E−02
ST3	7.208E−02	5.373E−02	4.213E−02	5.538E−02	5.970E−02	1.758E−01	5.460E−02
ST4	1.126E−02	8.395E−03	6.583E−03	8.654E−03	9.327E−03	2.747E−02	8.531E−03
ST5	1.352E−02	1.007E−02	7.899E−03	1.038E−02	1.119E−02	3.297E−02	1.024E−02
ST6	1.577E−02	1.175E−02	9.216E−03	1.212E−02	1.306E−02	3.846E−02	1.194E−02
ST7	2.253E−02	1.679E−02	1.317E−02	1.731E−02	1.865E−02	5.495E−02	1.706E−02
ST8	2.703E−02	2.015E−02	1.580E−02	2.077E−02	2.239E−02	6.593E−02	2.048E−02
ST9	3.154E−02	2.351E−02	1.843E−02	2.423E−02	2.612E−02	7.692E−02	2.389E−02
ST10	3.604E−02	2.686E−02	2.107E−02	2.769E−02	2.985E−02	8.791E−02	2.730E−02
ST11	4.055E−02	3.022E−02	2.370E−02	3.115E−02	3.358E−02	9.890E−02	3.071E−02
ST12	4.505E−02	3.358E−02	2.633E−02	3.462E−02	3.731E−02	1.099E−01	3.413E−02
ST13	5.181E−02	3.862E−02	3.028E−02	3.981E−02	4.291E−02	1.264E−01	3.924E−02
ST14	5.406E−02	4.030E−02	3.160E−02	4.154E−02	4.477E−02	1.319E−01	4.095E−02
ST15	6.307E−02	4.701E−02	3.686E−02	4.846E−02	5.223E−02	1.538E−01	4.778E−02
ST16	6.758E−02	5.037E−02	3.950E−02	5.192E−02	5.596E−02	1.648E−01	5.119E−02
ST17	7.208E−02	5.373E−02	4.213E−02	5.538E−02	5.970E−02	1.758E−01	5.460E−02

**Table 6a:** Average daily dose (ADD) via ingestion pathway for As concentration.

**As ADD**_**IN**_**values**							
*Station ID*	(6–12 months)	(6–11 years)	(11–16 years)	(16–18 years)	(18–21 years)	(≥21 years)	(>65 years)
ST1	4.066	1.667	1.242	0.974	1.281	1.380	1.263
ST2	4.066	1.667	1.242	0.974	1.281	1.380	1.263
ST3	5.824	2.388	1.780	1.396	1.835	1.977	1.809
ST4	5.824	2.388	1.780	1.396	1.835	1.977	1.809
ST5	5.385	2.208	1.645	1.290	1.696	1.828	1.672
ST6	5.165	2.117	1.578	1.238	1.627	1.754	1.604
ST7	4.396	1.802	1.343	1.053	1.385	1.492	1.365
ST8	3.626	1.487	1.108	0.869	1.142	1.231	1.126
ST9	3.516	1.442	1.075	0.843	1.108	1.194	1.092
ST10	3.407	1.397	1.041	0.816	1.073	1.157	1.058
ST11	3.736	1.532	1.142	0.895	1.177	1.269	1.160
ST12	4.945	2.027	1.511	1.185	1.558	1.679	1.536
ST13	3.407	1.397	1.041	0.816	1.073	1.157	1.058
ST14	4.615	1.892	1.410	1.106	1.454	1.567	1.433
ST15	6.264	2.568	1.914	1.501	1.973	2.127	1.945
ST16	6.703	2.748	2.048	1.606	2.112	2.276	2.082
ST17	8.352	3.424	2.552	2.001	2.631	2.836	2.594

In addition, the $RfD_{metal}$ measured in mg/kg/day stands for a maximum acceptable oral reference dose for a typical heavy metal varies for different metals. Table 2 presents the values of $RfD_{metal}$ for different metals obtained in this study [9], [10], [15]. Several literatures have established that a risk is very likely when the $HRI_{IN}$ is equal or greater than unity while the probability of a risk not occurring is postulated when $HRI_{IN}$ is less than unity [16], [17], [18], [19]. These values are tabulated in Table 3a, Table 3b, Table 4a, Table 4b, Table 5a, Table 5b, Table 6a, Table 6b, Table 7a, Table 7b(a)–(b) for all the metals obtained in this study.

**Table 6b:** Health Risk Index (HRI) via ingestion pathway for As concentration.

**As HRI**_**IN**_**values**							
*Station ID*	(6–12 months)	(6–11 years)	(11–16 years)	(16–18 years)	(18–21 years)	(≥21 years)	(>65 years)
ST1	5.556E+00	4.141E+00	3.248E+00	4.269E+00	4.602E+00	1.355E+01	4.209E+00
ST2	5.556E+00	4.141E+00	3.248E+00	4.269E+00	4.602E+00	1.355E+01	4.209E+00
ST3	7.959E+00	5.932E+00	4.652E+00	6.115E+00	6.591E+00	1.941E+01	6.029E+00
ST4	7.959E+00	5.932E+00	4.652E+00	6.115E+00	6.591E+00	1.941E+01	6.029E+00
ST5	7.358E+00	5.485E+00	4.301E+00	5.654E+00	6.094E+00	1.795E+01	5.574E+00
ST6	7.058E+00	5.261E+00	4.125E+00	5.423E+00	5.845E+00	1.722E+01	5.346E+00
ST7	6.007E+00	4.477E+00	3.511E+00	4.615E+00	4.975E+00	1.465E+01	4.550E+00
ST8	4.956E+00	3.694E+00	2.896E+00	3.808E+00	4.104E+00	1.209E+01	3.754E+00
ST9	4.805E+00	3.582E+00	2.809E+00	3.692E+00	3.980E+00	1.172E+01	3.640E+00
ST10	4.655E+00	3.470E+00	2.721E+00	3.577E+00	3.855E+00	1.136E+01	3.526E+00
ST11	5.106E+00	3.806E+00	2.984E+00	3.923E+00	4.228E+00	1.245E+01	3.868E+00
ST12	6.758E+00	5.037E+00	3.950E+00	5.192E+00	5.596E+00	1.648E+01	5.119E+00
ST13	4.655E+00	3.470E+00	2.721E+00	3.577E+00	3.855E+00	1.136E+01	3.526E+00
ST14	6.307E+00	4.701E+00	3.686E+00	4.846E+00	5.223E+00	1.538E+01	4.778E+00
ST15	8.560E+00	6.380E+00	5.003E+00	6.577E+00	7.089E+00	2.088E+01	6.484E+00
ST16	9.160E+00	6.828E+00	5.354E+00	7.038E+00	7.586E+00	2.234E+01	6.939E+00
ST17	1.141E+01	8.507E+00	6.671E+00	8.769E+00	9.452E+00	2.784E+01	8.645E+00

**Table 7a:** Average daily dose (ADD) via ingestion pathway for Pb concentration.

**Pb ADD**_**IN**_**values**							
*Station ID*	(6–12 months)	(6–11 years)	(11–16 years)	(16–18 years)	(18–21 years)	(≥21 years)	(>65 years)
ST1	ND	ND	ND	ND	ND	ND	ND
ST2	ND	ND	ND	ND	ND	ND	ND
ST3	ND	ND	ND	ND	ND	ND	ND
ST4	1.099	0.451	0.336	0.263	0.346	0.373	0.341
ST5	0.769	0.315	0.235	0.184	0.242	0.261	0.239
ST6	0.769	0.315	0.235	0.184	0.242	0.261	0.239
ST7	ND	ND	ND	ND	ND	ND	ND
ST8	ND	ND	ND	ND	ND	ND	ND
ST9	ND	ND	ND	ND	ND	ND	ND
ST10	ND	ND	ND	ND	ND	ND	ND
ST11	ND	ND	ND	ND	ND	ND	ND
ST12	ND	ND	ND	ND	ND	ND	ND
ST13	ND	ND	ND	ND	ND	ND	ND
ST14	ND	ND	ND	ND	ND	ND	ND
ST15	ND	ND	ND	ND	ND	ND	ND
ST16	ND	ND	ND	ND	ND	ND	ND
ST17	ND	ND	ND	ND	ND	ND	ND

**Table 7b:** Health Risk Index (HRI) via ingestion pathway for Pb concentration.

**Pb HRI**_**IN**_**values**							
*Station ID*	(6–12 months)	(6–11 years)	(11–16 years)	(16–18 years)	(18–21 years)	(≥21 years)	(>65 years)
ST1	ND	ND	ND	ND	ND	ND	ND
ST2	ND	ND	ND	ND	ND	ND	ND
ST3	ND	ND	ND	ND	ND	ND	ND
ST4	3.218E−01	2.399E−01	1.881E−01	2.473E−01	2.665E−01	7.849E−01	2.438E−01
ST5	2.253E−01	1.679E−01	1.317E−01	1.731E−01	1.865E−01	5.495E−01	1.706E−01
ST6	2.253E−01	1.679E−01	1.317E−01	1.731E−01	1.865E−01	5.495E−01	1.706E−01
ST7	ND	ND	ND	ND	ND	ND	ND
ST8	ND	ND	ND	ND	ND	ND	ND
ST9	ND	ND	ND	ND	ND	ND	ND
ST10	ND	ND	ND	ND	ND	ND	ND
ST11	ND	ND	ND	ND	ND	ND	ND
ST12	ND	ND	ND	ND	ND	ND	ND
ST13	ND	ND	ND	ND	ND	ND	ND
ST14	ND	ND	ND	ND	ND	ND	ND
ST15	ND	ND	ND	ND	ND	ND	ND
ST16	ND	ND	ND	ND	ND	ND	ND
ST17	ND	ND	ND	ND	ND	ND	ND
