# Supplementary material for: Computation of human health risk in surface water in Ado-Odo Ota, Ogun State, Nigeria
Source: Data Brief. 2018 Jun 23;19:1574–83. doi: 10.1016/j.dib.2018.06.051 (PMC6141265; doi:10.1016/j.dib.2018.06.051)
Supplement: Supplementary file 1 — Supplementary material [file mmc1.docx]

TENEBE IMOKHAI THEOPHILUS

*Department of Civil Engineering, Covenant University, Ota, Ogun State, Nigeria.*

Email: [yoshearer@gmail.com, imokhai.tenebe@covenantuniversity.edu.ng](mailto:yoshearer@gmail.com,%20imokhai.tenebe@covenantuniversity.edu.ng)

**June 10, 2018**

**Data In Brief**

Dear Editor,

The authors declare no conflict of interest.

*Signatures and names of all the authors*

**TENEBE I.T.**

**Corresponding Author on behalf of co-authors:**

**Ogbiye A.S.**

**Emenike P.C**

**Anake U.W.**
